# Phosphorus dynamics in litter–soil systems during litter decomposition in larch plantations across the chronosequence

**DOI:** 10.3389/fpls.2022.1010458

**Published:** 2022-10-07

**Authors:** Guangyu Chi, Fanpeng Zeng, Yang Wang, Xin Chen

**Affiliations:** ^1^ Key Laboratory of Pollution Ecology and Environment Engineering, Institute of Applied Ecology, Chinese Academy of Sciences, Shenyang, China; ^2^ College of Life Science, Nanchang Normal University, Nanchang, China; ^3^ Northeast Institute of Geography and Agroecology, Chinese Academy of Sciences, Changchun, China

**Keywords:** stand age, nutrient dynamic, phosphorus fraction, forest soil, litter

## Abstract

The dynamics of phosphorus (P) in litter–soil systems during litter decomposition across a plantation chronosequence remain to be underinvestigated, especially in terms of the nutrient cycle in plantations. In this study, the P dynamics in a litter–soil system of larch (*Larix kaempferi*) plantations at three stand ages (10, 25, and 50 years old) were examined through a 4-year *in situ* decomposition experiment (experiment 1) and a 360-day indoor incubation experiment (experiment 2). The aim of experiment 1 and experiment 2 is to determine the P dynamics in litter and soil, respectively. The results in experiment 1 suggested that litter mass retained 34.1%–42.5% of the initial mass after a 4-year decomposition period, and the turnover time (t_0.95_) of the decomposition was 11.3, 13.9, and 11.8 years for 10-, 25- and 50-year-old stand larch plantations, respectively. Litter exhibited a net P decrease during the first 180 days, followed by a phase of a net P increase. The lowest P accumulation rate was found in the 25-year-old stand during the P immobilization stage. This immobilization phase was followed by a slow litter P decrease. Highly correlated relations were found between the litter decomposition rate and the initial litter N concentration and C/N, whereas the P accumulation rate was noticeably correlated with the initial litter P and C/P. The results in experiment 2 showed that litter addition promoted the accumulation of the highly labile P (resin P, NaHCO_3_-P_i_, and NaHCO_3_-P_o_), as well as moderately labile P_i_ (NaOH-P_i_) in the soil. The findings obtained suggest that soil microbial biomass P and acid phosphatase activity were the primary factors driving the activation of soil P during litter decomposition. These findings would be beneficial to the systematic understanding of the nutrient cycle in plant–soil systems and litter management during the development of larch plantations.

## Introduction

Phosphorus (P) is a critical nutrient in forest soil (Rui et al., 2012), and its availability affects ecosystem functions such as soil carbon (C) storage (Fisk et al., 2015), nitrogen (N) fixation (Vitousek and Howarth, 1991), microbial respiration (Spohn and Schleuss, 2019), and litter decomposition (McDonald et al., 2013). In recent years, more and more studies showed that temperate forest ecosystems have been limited by P (Braun et al., 2010; Hou et al., 2012; Shaw and DeForest, 2013; Yan et al., 2018).

Natural secondary forests are widely distributed over temperate parts of the world (Wang and Wang, 2007), which have been formed from primary forests after extreme natural events or intense human activities (Yang et al., 2010a). Roughly 70% of the forest in Northeast China comprises secondary forest (Zhu et al., 2019). However, the secondary forests cannot satisfy the increasing need for timber and other forest products. Since the 1960s, a large number of secondary forests were converted to larch (*Larix* spp. including *L. kaempferi, L. olgensis*, and *L. principis-rupprechtii*) plantations for fast timber production (Yang et al., 2010b; Zhu et al., 2019). Meanwhile, a decrease in soil nutrients has occurred in these areas because of the mono-silviculture system and unreasonable harvesting and thinning regimes of larch plantations (Yang et al., 2013; Yan et al., 2017; Zheng et al., 2017a). Among the declined nutrient elements, P has been considered to be a limited factor that influences the growth of larch with forest aging (Chen et al., 2016).

Plant litter has a vital role in maintaining soil fertility by regulating the nutrient cycle during decomposition (Zhang et al., 2016). As a fundamental process driving global C and nutrient cycling in terrestrial ecosystems, litter decomposition has been proven to contribute up to 75%–95% of P supply to plants (Nordén, 1994; Pourhassan et al., 2016). Litter decomposition is generally driven by several factors, namely, climate, litter quality, and soil properties. Climate is the primary controlling factor of litter decomposition on a global scale, attributable to the biological process during decomposition being significantly regulated by temperature and moisture. However, plant litter decomposition is determined by litter quality, soil physicochemical, and microbial properties on a regional scale (Zhang et al., 2016). As plants develop, the quantity and quality of forest litter, as well as the soil physicochemical and microbiological properties, change accordingly (Chen et al., 2018; Liu et al., 2018; Paz-Ferreiro et al., 2018). This may lead to changes in the process of litter decomposition and nutrient release. More knowledge about the dynamic changes of litter P with plant age would allow for a better understanding of the nutrient cycle during the development of larch plantations. This has beneficial implications for nutrient conservation and litter management in larch plantations. Although numerous studies have focused on P cycling in plantation ecosystems of various ages (Zheng et al., 2017a; Paz-Ferreiro et al., 2018; Yan et al., 2018; Zeng et al., 2018; Wang et al., 2019), little is known about the P dynamics in litter–soil systems during litter decomposition across a plantation chronosequence. This would be of great help for a systematic understanding of the nutrient cycle during forest development.

To investigate the P dynamics in litter–soil systems during litter decomposition across the larch plantation chronosequence, we collected plant litter and soil samples from larch (*L. kaempferi*) plantations at three stand ages (10, 25, and 50 years old). Then, a 4-year *in situ* decomposition experiment (experiment 1) and a 360-day indoor incubation experiment (experiment 2) were conducted. The purpose of experiment 1 and experiment 2 was to determine the P dynamics in litter and soil, respectively. We hypothesized that (1) forest age would affect the properties of litter, thus leading to a difference in the process of litter decomposition and P dynamics, and (2) litter additions would change soil physicochemical properties, thus affecting the activation of soil P.

## Materials and methods

### Study site

This study was conducted in larch plantations located on the Qingyuan Forest Chinese Ecosystem Research Network, Chinese Academy of Sciences (41°51′N, 124°54′E). The climate in this area falls under the continental monsoon type, which is humid and rainy during summer and cold and snowy during winter. The mean annual air temperature varies from 3.9°C to 5.4°C, and the maximum and minimum temperatures are 36.5°C and −37.6°C in July and January, respectively. The annual rainfall is 700–850 mm, with over 80% occurring from June to August. The soil type in the region is brown forest soil based on USDA soil taxonomy, and the soil freeze–thaw cycle occurs from November to the following April (Zeng et al., 2018).

This region was originally occupied by a mixed Korean pine broad-leaved forest, and it was converted to a secondary broad-leaved forest because of a fire in the early 1950s. Since the 1960s, the natural secondary forest was replaced by larch plantations (Wang et al., 2020).

### Plant litter and soil sampling

We selected three even-aged and pure larch (*L. kaempferi*) plantation stands (10, 25, and 50 year old) within the Qingyuan Forest Chinese Ecosystem Research Network, Chinese Academy of Sciences, to represent a chronosequence. Three plantation areas were chosen for each of three forest ages on the slightly variable slopes and altitudes to avoid the difference caused by topographical properties.

When the needles of the larch plantation began to senesce in late September 2015, we installed 10 litter collectors (1 m × 1 m) in each plantation area to obtain the litter samples (**Figure S1A**). In November 2016, the litter samples were collected and transported to the laboratory and air-dried at room temperature. The samples were weighted to calculate the amount of litter per unit area and then divided into three parts. One subsample was ground and sieved for the determination of its C, N, and P contents. Another set of samples was used to conduct an indoor incubation experiment. The remaining samples were used to undertake an *in situ* decomposition experiment.

Five soil samples with a 0- to 10-cm layer were extracted from each plantation area and mixed into one soil sample. The soil samples were then divided into two subsamples after being air-dried and ground. One subsample was used to determinate the physicochemical properties of the soil. The remaining samples were used to perform an indoor incubation experiment to simulate the P dynamics of soil. The general properties of the litter and soil in different-aged stands of larch plantations are shown in **Table 1**.

**Table 1:** General properties of litter and soil in different-aged stands of larch plantation.

Stand age (year)	General properties					
	Soil (g kg^−1^)			Litter (g kg^−1^)		
	TC	TN	TP	C	N	P
10	24.39 ± 2.49b	1.29 ± 0.06a	0.53 ± 0.01a	476.71 ± 12.67a	8.35 ± 0.06a	0.34 ± 0.04b
25	30.44 ± 1.90a	1.47 ± 0.09a	0.49 ± 0.01b	479.34 ± 16.33a	6.82 ± 0.15c	0.49 ± 0.05a
50	31.52 ± 2.09a	1.44 ± 0.08a	0.33 ± 0.02c	483.76 ± 17.13a	7.68 ± 0.23b	0.22 ± 0.03c

Values are means ± SE (n = 3). Same letters represent no statistically significant difference among stand ages (p< 0.05). TC, total carbon; TN, total nitrogen; TP, total phosphorus; C, carbon; N, nitrogen; P, phosphorus.

### Plant litter decomposition experiments

Experiment 1: An *in situ* decomposition experiment was performed using a nylon mesh (0.5 mm) litterbag (10 cm × 10 cm) filled with a 10-g litter sample of larch (**Figure S1B**). In November 2016, 20 litterbags were placed on the forest floor in each plantation area. In order to observe the dynamics of litter P for 1 year, one litterbag in each plantation area was collected at 180, 240, and 300 days postincubation. After that, the litterbags were collected at 1, 1.5, 2, 2.5, 3, 3.5, and 4 years, with the aim to monitor the annual variation of P. The collected litter was dried, weighed, and sieved to determine the concentration of C, N, and P.

Experiment 2: An indoor incubation experiment was conducted in 300-ml glass bottles. Two treatments (litter addition and control) were set for each of the three forest ages, with three replicates for each treatment. For the litter addition treatment, 100 g of air-dried and sieved (<2 mm) soil and corresponding dried litter were placed into bottles (**Table S1**). The amount of litter to be added was determined by the annual amount of litter. For the control group, only soil was applied. The polyethylene film with pinholes was applied to cover the bottles, and soil samples were incubated at 60% water holding capacity by the weight method at 25°C. The soil moisture content was maintained by regularly reweighing and adding water. Three bottles from each treatment were destructively sampled on days 0, 30, 60, 120, 240, and 360. The samples were divided into two subsamples after the litter was picked out. One subsample was used to determinate the acid phosphatase activity (APA) and microbial biomass P (MBP). The remaining soil samples were oven-dried, ground, and sieved to determine the total C (TC), total N (TN), total P (TP), and P fractions.

### Analytical methods

The concentrations of C and N in the litter and soil were analyzed using an elemental analyzer (Elementar Vario Macro cube, Germany). The concentrations of P in litter and soil were extracted by concentrated H_2_SO_4_ and determined by molybdenum blue colorimetry. MBP of the soil was analyzed using the fumigation extraction method (Chodak et al., 2021). The APA was measured by the disodium *p*-nitrophenyl phosphate method (Hou et al., 2015).

Soil P fractions were measured using a modified Hedley fraction method, in which the inorganic P (P_i_) and/or organic P (P_o_) of decreasing activity was removed by sequential extraction (Hedley et al., 1982; Zeng et al., 2018). The resin P was extracted by the anion exchange resin. The NaHCO_3_-P_i_ and NaHCO_3_-P_o_ were extracted using 0.5 mol L^−1^ NaHCO_3_. The NaOH-P_i_ and NaOH-P_o_ were extracted by 0.1 mol L^−1^ NaOH. The HCl-P_i_ was extracted by 1 mol L^−1^ HCl. Afterwards, the residual P was digested with concentrated H_2_SO_4_ and HClO_4_ and then measured using the ascorbic acid molybdenum blue method (Wang et al., 2006). Other soil P fractions were measured using a malachite green method (Zhao et al., 2013). According to the nutrient availability to plant, P fractions were divided into soil highly labile P (resin P, NaHCO_3_-P_i_, and NaHCO_3_-P_o_), moderately labile P (NaOH-P_i_ and NaOH-P_o_), stable P (HCl-P_i_), and residual P (Hedley et al., 1982).

### Data analyses

The remaining litter mass and P were calculated using the following equations:


(1) $$\text{Net mass remaining }({\text{t hm}}^{-2})=\left({\text{W}}_{\text{t}}/{\text{W}}_{0}\right)\times {\text{Q}}_{\text{n}}$$

(2) $$\text{Net P remaining }({\text{kg hm}}^{-2})=\left({\text{C}}_{\text{t}}{\text{W}}_{\text{t}}/{\text{W}}_{0}\right)\times {\text{Q}}_{\text{n}}\times 10^{-3}$$

(3) $$\text{Relative mass remaining}\left(\%\right)=\left({\text{W}}_{\text{t}}/{\text{W}}_{0}\right)\times 100\%$$

(4) $$\text{Relative P remaining}\left(\%\right)=\left({\text{C}}_{\text{t}}{\text{W}}_{\text{t}}\right)/\left({\text{C}}_{0}{\text{W}}_{0}\right)\times 100\%$$

(5) $$\text{P accumulation rate}=({\text{C}}_{\text{max}}{\text{W}}_{\text{max}}–{\text{C}}_{0}{\text{W}}_{0})/{\text{t}}_{\text{max}}$$

where Q_n_ (t hm^−2^) is the total amount of litter produced each year in the unit area of n-year forest age; W_0_ (g) and C_0_ (g kg^−1^) are the initial oven-dried weight per litterbag and P concentration of litter, respectively; W_t_ (g) and C_t_ (g kg^−1^) are the oven-dried weight per litterbag and P concentration of litter at a specific time t, respectively; and W_max_, C_max_, and t_max_ are the oven-dried weight, P concentration, and time when the relative P remaining reaches the maximum, respectively.

The mass remaining of litter was expressed using the follow equation:


(6) $${\text{W}}_{\text{t}}={\text{W}}_{0}{\text{e}}^{-\text{kt}}$$

where k and t represent the decomposition rate constant (year^−1^) and decomposition time, respectively.

Primary and interaction effects of stand ages, litter addition, and incubation time were assessed by a general linear model. Stand ages, litter addition, and incubation time used in the general linear model were categorical predictors. The effect of litter addition on soil P fractions was determined by one-way ANOVA. A difference at the *p*< 0.05 level was considered statistically significant. The statistics were analyzed using SPSS 19.0.

Redundancy analysis (RDA) was conducted to identify the dominant factors influencing the dynamics of P fractions using Canoco 5.0 software. A Monte Carlo test was employed to estimate the significance of the correlation between the ordination and explanatory variables. A forward selection method was applied to evaluate the responses of soil P fractions to soil environmental factors.

Structural equation modeling (SEM) was employed to better understand how the dominant factors directly or indirectly affect the soil P fractions. A multivariate functional index, labile P, was created to represent resin P, NaHCO_3_-P_i_, NaHCO_3_-P_o_, NaOH-P_i_, and NaOH-P_o_ by principal component analysis prior to the SEM procedure. Then, the first principal components, which explained 65% of the variations in the original variables, were used in the subsequent SEM analysis. The goodness of model fit was identified by a high comparative fit index (CFI > 0.9), nonsignificant chi-square test (*p* > 0.05), and low root-mean-square errors of approximation (RMSEA< 0.05). SEM analysis was performed using AMOS 24.0 (Fan et al., 2022).

## Results

### Mass loss and decomposition rate

**Figure 1:**
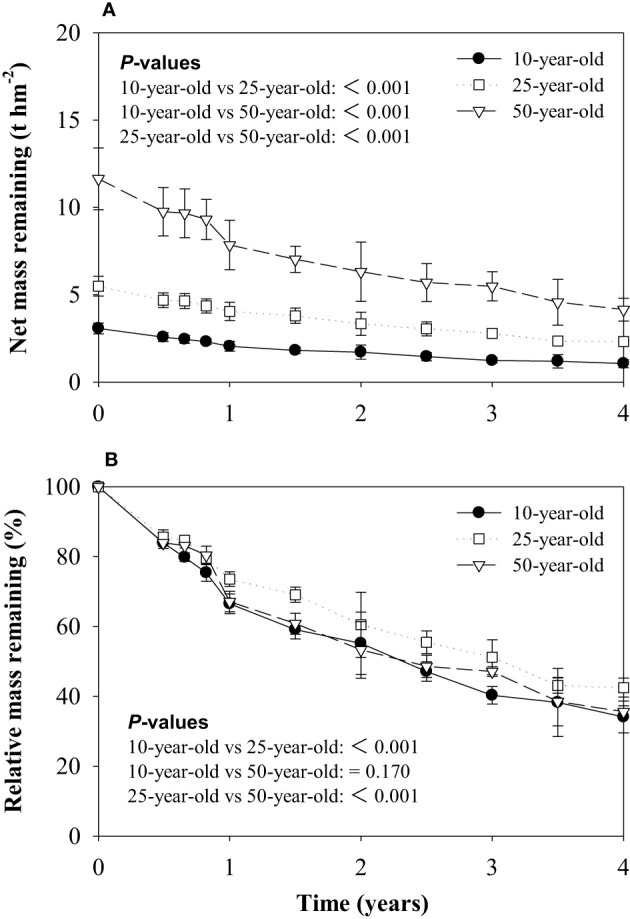
Net mass remaining **(A)** and relative mass remaining **(B)** of litter during decomposition in experiment 1. *P*-values for effects of stand ages are obtained by linear mixed model.

The net mass remaining of the 50-year-old stand was the highest, followed by the 25-year-old stand, and both masses were noticeably higher than the 10-year-old stand (**Figure 1A**). The litter relative mass retained 34.1%–42.5% of the initial mass after a 4-year decomposition period, and the highest litter mass remaining was found in the 25-year-old stand. The variation of the remaining biomass in the three forest ages was characterized by an initial phase of rapid decrease (14.4%–16.1% during the first 180 days), followed by a second and a slightly slower phase (**Figure 1B**).

The dynamics of litter mass loss for the larch plantations accorded with the exponential model (*R* ^2^ > 0.97). No marked difference in the litter decomposition rate constant (k value) was found between the 10- and 50-year-old stands, but a significantly lower k value (0.208 year^−1^) was exhibited in the 25-year-old stand. The turnover time (t_0.95_) of the decomposition for 10-, 25-, and 50-year-old larch plantations was 11.3, 13.9, and 11.8 years, respectively (**Table 2**).

**Table 2:** Decomposition rate constants (k values) and turnover time (t_0.95_) for litter in experiment 1.

Stand age (year)	k (year^−1^)	t_0.95_ (year)	*SE*	*R* ^2^	*p*	*n*
10	0.265	11.3	0.03	0.983	***	11
25	0.215	13.9	0.03	0.988	***	11
50	0.254	11.8	0.04	0.975	***	11

R^2^, coefficient of determination; SE, standard error. ***indicates significance at p< 0.001.

### Phosphorus dynamics in litter during decomposition

**Figure 2:**
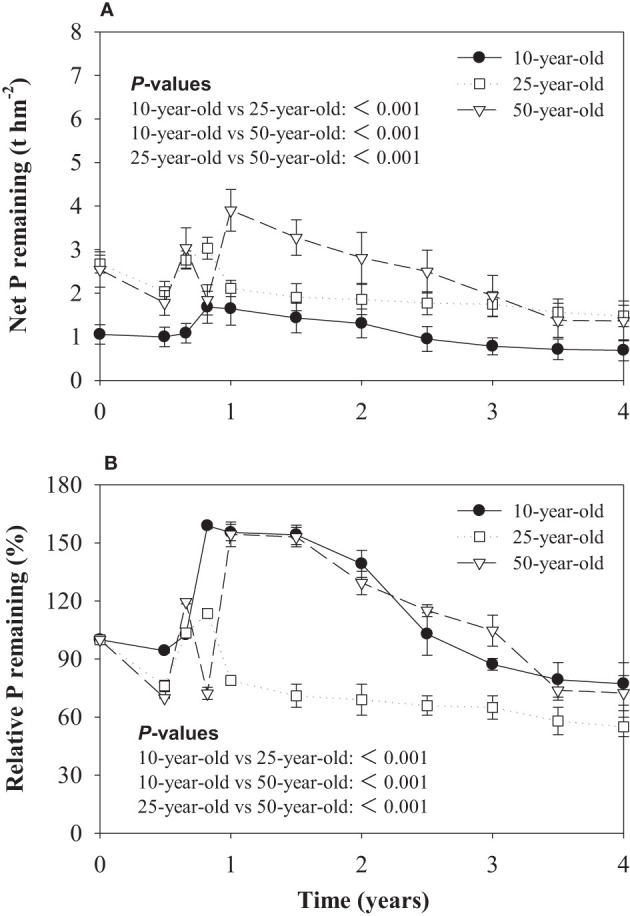
Litter net phosphorus (P) remaining **(A)** and relative phosphorus (P) remaining **(B)** over the decomposition period in experiment 1. *P*-values for effects of stand ages are obtained by linear mixed model.

Similar to the litter net mass remaining, the lowest change in net P remaining was found in the 10-year-old stand (**Figure 2A**). The litter of the three differently age larch plantations exhibited a decrease in P during the first 180 days of the decomposition period. The P remaining of larch litter in 10-, 25-, and 50-year-old stands were 94.3%, 76.1%, and 69.9%, respectively. After 180 days, the P remaining of litter increased, and the lowest P accumulation rate was found in the 25-year-old stand. This phase was followed by a period of slow release during the late stage of litter decomposition (**Figure 2B**).

**Figure 3:**
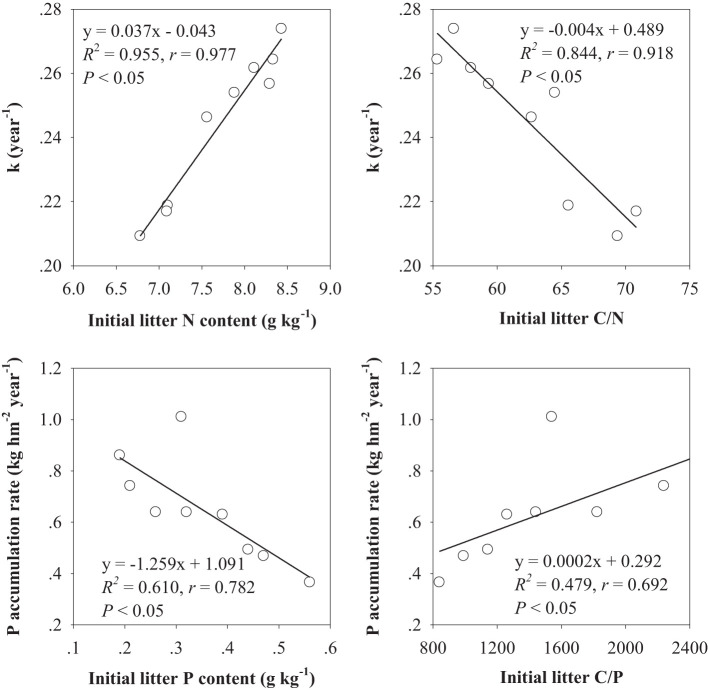
Relationship between dynamics of litter mass and P and initial leaf litter quality in experiment 1; n = 9.

The results of correlation analysis showed that the k value was positively correlated with the initial litter N and negatively correlated with the initial litter C/N. The P accumulation rate was markedly negatively correlated with the initial litter P and positively correlated with the initial litter C/P (**Figure 3**).

### Phosphorus dynamics in soil during decomposition

**Figure 4:**
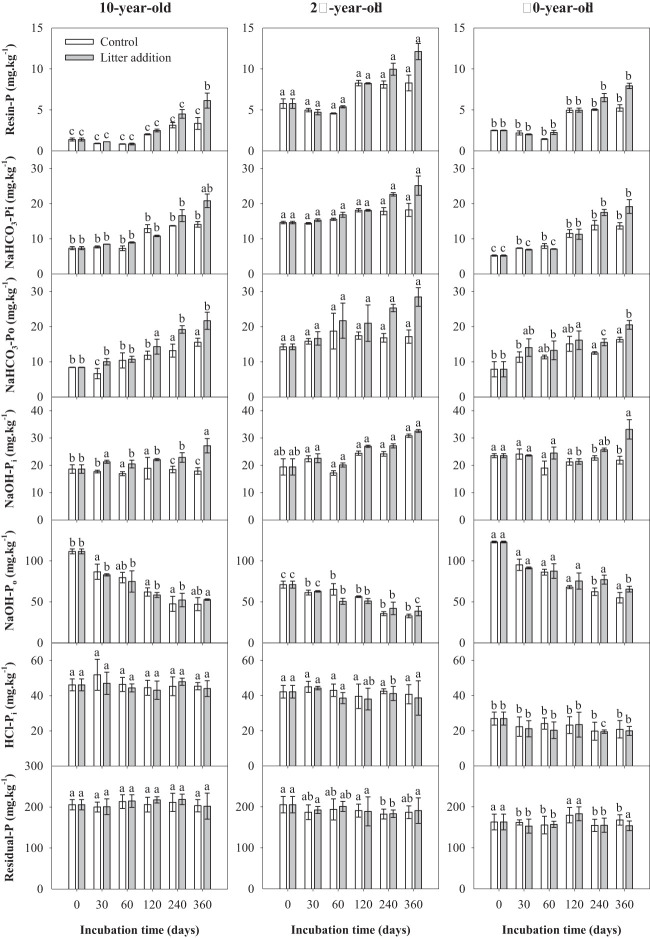
Variations of soil phosphorus (P) fractions over the incubation period in experiment 2. Same letters represent no statistically significant differences among stand ages in the same treatment and incubation time (*p*< 0.05).

During indoor incubation, all soil P fractions were found to be sensitive to stand age. Both highly labile P (resin P, NaHCO_3_-P_i_, and NaHCO_3_-P_o_) and moderately labile P_i_ (NaOH-P_i_) showed a positive response to litter addition and incubation time. With the exception of moderately labile P_o_ (NaHCO_3_-P_o_), all labile P were influenced significantly by stand age × incubation time and litter addition × incubation time interactions (**Table 3**). The addition of litter promoted the accumulation of soil highly labile P and moderately labile P_i_. During soil incubation, the soil moderately labile P_o_ showed a decrease in both control and litter addition treatments. Both the contents of stable and residual P did not significantly change during the incubation period (**Figure 4**).

**Table 3:** Effects of stand age, litter addition, and incubation time on soil P in experiment 2.

Soil P fractions	Effects						
	Stand age (*S*)	Litter addition (*L*)	Incubation time (*I*)	*S* × *L*	*S* × *I*	*L* × *I*	*S* × *L* × *I*
Resin P	***	***	***	ns	***	***	ns
NaHCO_3_-P_i_	***	***	***	ns	***	***	ns
NaHCO_3_-P_o_	***	***	***	ns	ns	**	ns
NaOH-P_i_	***	***	***	ns	***	***	*
NaOH-P_o_	***	ns	***	ns	***	*	ns
HCl-P_i_	***	ns	ns	ns	ns	ns	ns
Residual P	***	ns	ns	ns	ns	ns	ns

*, **, and *** indicate significance at p< 0.05, p< 0.01, and p< 0.001, respectively. P, phosphorus; ns, not significant.

**Figure 5:**
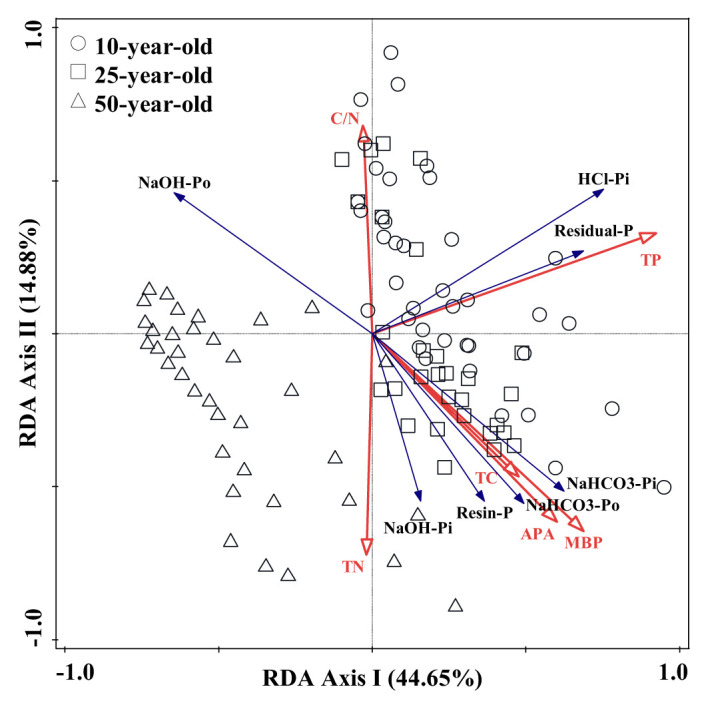
Redundancy analysis (RDA) on the relationships between soil phosphorus (P) fractions and environmental factors under different treatments in experiment 2. Blue and red arrows represent soil P fractions and environmental factors, respectively. The length and angle of lines indicate relationships between soil P fractions, environmental factors, and principal component analysis (PCA) axes; n = 108. Abbreviations: TP, total phosphorus; MBP, microbial biomass P; APA, acid phosphatase activity; TC, total carbon; TN, total nitrogen; C/N, total carbon/total nitrogen.

The results of RDA revealed that the first and second component axes explained approximately 44.65% and 14.88% of the total variation of P fractions, respectively. Meanwhile, the first two axes retained 98.52% of cumulative variance, which originated in the correlation between soil environmental factors and P fractions (**Table 4**). This indicates that this correlation was well represented by these first two axes. The correlation was further elucidated by a triplot, which suggested that TP, MBP, APA, and C/N had a greater impact on the dynamics in soil P fractions in the larch plantation (**Figure 5**). The partial Monte Carlo test was applied to estimate the contribution of soil environmental factors to the changes in soil P fractions (**Table S2**). The results suggest that the order of contribution was as follows: TP > MBP > APA > C/N > TN > TC. The effects of TP, MBP, APA, and C/N on the soil P fractions were remarkable, with TP, MBP, APA, and C/N explaining 39.6%, 15.9%, 2.2%, and 2.2% of the difference between soil P fractions, respectively.

**Table 4:** Soil P fractions in relation to soil environmental factors in experiment 2.

Axes	I	II	III	IV
Eigenvalues	0.447	0.149	0.007	0.002
Soil P fractions–soil environmental factors correlations	0.842	0.697	0.645	0.219
Percentage variance of soil P fractions (%)	44.65	14.88	0.71	0.16
Cumulative percentage variance of soil P fractions–soil environmental factors correlations (%)	73.89	98.52	99.68	99.95

P, phosphorus.

**Figure 6:**
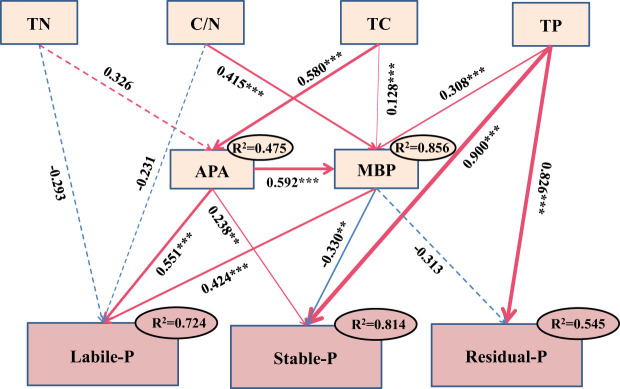
Structural equation modeling (SEM) analysis for effects of environmental factors on soil phosphorus (P) fractions in experiment 2. Goodness-of-fit statistics for the model are as follows: *χ* ^2^ = 0.569, *p* = 0.904, CFI = 1.000, RMSEA = 0.000, AIC = 1829.317. ** and *** indicate *p*< 0.01 and *p*< 0.001. Red and blue lines indicate positive and negative relationships, respectively. Arrows suggest the hypothesized direction of causation. Solid lines indicate significant pathways, whereas dashed lines indicate nonsignificant pathways. The standardized path coefficients are indicated by values adjacent to the arrows. Arrow thickness is proportional to the relationship strength. *R* ^2^ values referred to the proportions of the observed variance; n = 108. Abbreviations: TP, total phosphorus; MBP, microbial biomass P; APA, acid phosphatase activity; TC, total carbon; TN, total nitrogen; C/N, total carbon/total nitrogen. Soil labile P refers to the first component (PC1) from the principal component analysis (PCA) conducted for resin P, NaHCO_3_-P_i_, NaHCO_3_-P_o_, NaOH-P_i_, and NaOH-P_o_.

The results of SEM indicated that labile P was positively correlated with MBP and APA, with the standardized coefficients being 0.424 (*p*< 0.001) and −0.551 (*p*< 0.001), respectively. Both stable and residual P were more sensitive to TP, with the standardized coefficients being 0.900 (*p*< 0.001) and 0.826 (*p*< 0.001), respectively. TC might have an indirect effect on soil labile and stable P through influencing MBP and APA. The combination of selected soil environmental factors explained 72.4%, 81.4%, and 54.5% of the variance for soil labile, stable, and residual P, respectively (**Figure 6**).

## Discussion

### Mass loss and decomposition rate

In this study, the changes in litter mass demonstrated obvious temporal patterns during decomposition. The mass loss of litter was higher during the early stage and was subsequently reduced. A similar finding was also reported in prior studies on forest ecosystems (Han et al., 2020). This could be attributed to the combined effects of the litter substrate quality and external environmental factors. In the first 180 days, abundant soil water after snow melting promotes soil microbial activity, and this is thus conducive to litter decomposition, because the litter quality is better during the early stages (Dziadowiec, 1987). As litter decomposition progresses, the ratio of recalcitrant substances gradually increases, resulting in a lower decomposition rate during the later stages (Matocha and Coyne, 2007).

We detected that the litter decomposition constant (k value) was markedly related to the initial litter N content and C/N ratio, indicating that the quality of litter in different forest ages was the key factor in influencing its decomposition rate. The litter of the 25-year-old larch had the lowest N content and the highest C/N (**Table 1**), which was not conducive to the growth and reproduction of soil microorganisms, resulting in a slow litter decomposition rate (Zhang et al., 2016). These results are consistent with those reported by prior studies (Gosz et al., 1973; Zheng et al., 2017b). Therefore, the N and C/N could be considered ideal susceptibility indicators of litter decomposition (Mafongoya et al., 2000; Gómez-Muñoz et al., 2014).

### Phosphorus dynamics in litter during decomposition

P remaining in litter showed a downward trend during the initial phases of decomposition, which were consistent with the general release pattern of P described by prior studies (Colpaert and Tichelen, 1996; Pourhassan et al., 2016). We speculate that this is caused by soluble P leaching from the litter during snow melting and rainfall. However, the litter exhibited a relatively low P decrease rate during the first 180 days of decomposition, and there was almost no change in P remaining in the litter of the 10-year-old forest during this period. This could be explained by the fact that coniferous litter is rich in recalcitrant compared with labile, more readily leachable compounds (José and Francisco, 2000). Although the content of P in the labile fraction was not determined directly, the results reported suggest that it might be a minor component of litter, especially in the 10-year-old larch.

After 180 days, a phase of a net P increase was observed, which could be attributed to the effects of microbial immobilization of P in the residual larch litter (Matocha and Coyne, 2007). One of the possible mechanisms underlying this immobilization was the retention of P in microbial biomass. A difference was observed in the initial element content of litter from different plants, which can mirror the combined effects from the element composition of leaves and nutrient retranslocation before the leaves fall (He et al., 2017). The pine species produce similar, low-quality needle litter with a low level of N, P, and other elements. Because of the lower C relative to P in decomposers, litter during this period contained an insufficient amount of P for the decomposers, which might lead to the accumulation of nutrients in litter (José and Francisco, 2000). This could be indicated by the highly correlated relations between the P accumulation rate and the initial litter P content and C/P ratio. Additionally, P immobilization might also be contributed by the translocation of P from fungal hyphae (Gosz et al., 1973).

The above P immobilization phase was followed by a slow P decrease period across all three treatments. The annual average release rates estimated for this liberation period ranged from 16.2% to 17.7%, and the low decay rate of litter might contribute to the release process of P. This seems to be a common trend, because such a phase characterized by a low P decrease rate during litter decomposition has also been reported in prior studies (King et al., 1997; Fujii and Takeda, 2010; Gómez-Muñoz et al., 2014).

### Phosphorus dynamics in soil during decomposition

Our results indicate that the content of soil TP gradually decreased with an increase in forest age (**Table 1**); this is consistent with prior studies (Ren et al., 2017; Liu et al., 2019). This phenomenon may be attributed to the following reasons. As the larch grows, P was absorbed and transported from the soil to aboveground, and the output of soil P continuously increased. Litter decomposition is the primary supplement of P in forest soil. With an increase in forest age, the litter amount gradually increased, and more P was stored in the litter.

The addition of litter was found to promote the accumulation of the highly labile P and the moderately labile P_i_, but a decrease in the content of the moderately labile P_o_ was reported during soil incubation. During soil incubation, the increase of soil highly labile P and moderately labile P_i_ might be due to the P release of litter. In addition, it also partly came from the mineralization of soil moderately labile P_o_. Prior studies found that during the mineralization process of moderately labile P_o_ in forest soil, some of them were converted into soil highly labile P to maintain the rapid growth of plants, whereas others were mineralized and fixed by the soil as moderately labile P_i_ (Liu et al., 2019). This is consistent with our results and indicates that moderately labile P_o_ in forest soil has an important P buffering role in the process of nutrient cycling.

RDA and SEM analysis indicated that TP, MBP, and APA were the dominant factors affecting the dynamics of soil P components during soil incubation. Soil P fractions were shown to be markedly influenced by TP, which has a significant positive correlation with stable and residual P. This is because these two P fractions accounted for a large part of TP and changed little during litter decomposition (**Figure 4**). The results suggested that soil stable and residual P were hard to activate, even in the case of litter addition. Soil labile P was positively correlated with MBP and APA (**Figure 6**). This is in accordance with a prior study, which has reported that P dynamics might be largely affected by microbial processes in forest soil (Bünemann et al., 2016). These processes include the mineralization of P_o_ inside and outside microbial cells (Spohn and Kuzyakov, 2013). MBP was used to quantify concentrations of P held within soil microbial cells, which play a significant role in P cycling by acting as a source or sink of P_i_ (Achat et al., 2010; Roberts et al., 2013). Outside the cells, acid phosphatase secreted by soil microorganisms plays a critical role in the cycling of soil P and determines the availability of this element (Vincent et al., 1992; Li et al., 2002; Chodak et al., 2021). The findings of this study suggested that soil MBP and APA were the primary factors driving the activation of soil P during litter decomposition.

## Conclusion

This study investigated the P dynamics in the litter–soil system of larch (*L. kaempferi*) plantations at three stand ages (10, 25, and 50 years old). The litter of the three differently age larch plantations exhibited a P decrease trend during the first 180 days of the decomposition period. After 180 days, the P remaining of litter increased, and the lowest P accumulation rate was found in the 25-year-old stand. This phase was followed by a period of slow release during the later stages of litter decomposition. During soil incubation, litter addition promoted the accumulation of the highly labile P and the moderately labile P_i_, whereas soil moderately labile P_o_ showed a decrease in both control and litter addition treatments. The results obtained suggest that soil MBP and APA were the primary factors driving the activation of soil P during litter decomposition. Litter decomposition is the primary supplement of P in forest soil. However, the decomposition speed of larch litter is relatively slow, and it takes a long time for P in litter to return to the forest soil. Therefore, litter should be prevented from being removed during the management of larch plantations.

## Data availability statement

The raw data supporting the conclusions of this article will be made available by the authors, without undue reservation.

## Author contributions

XC and GC conceived and designed the experiments. GC and FZ performed the data acquisition, analyzed the data and wrote the manuscript. XC and YW commented and edited the article. All authors contributed to the article and approved the submitted version.

## Acknowledgments

The authors gratefully acknowledge the National Natural Science Foundation of China (31470624) and the Strategic Priority Research Program of the Chinese Academy of Sciences (XDA23070502).

## Conflict of interest

The authors declare that the research was conducted in the absence of any commercial or financial relationships that could be construed as a potential conflict of interest.

## Publisher’s note

All claims expressed in this article are solely those of the authors and do not necessarily represent those of their affiliated organizations, or those of the publisher, the editors and the reviewers. Any product that may be evaluated in this article, or claim that may be made by its manufacturer, is not guaranteed or endorsed by the publisher.

## Supplementary material

Supplementary Table 1 Amount of litter addition (g.100 g soil^−1^) in experiment 2.

Supplementary Table 2 Importance and significance level of environmental factors in experiment 2.

Supplementary Figure 1 Litter collection **(A)** and litterbag experiment **(B)**.
