# Supplementary material for: Phosphorus dynamics in litter–soil systems during litter decomposition in larch plantations across the chronosequence
Source: Front Plant Sci. 2022 Oct 7;13:1010458. doi: 10.3389/fpls.2022.1010458 (PMC9585294; doi:10.3389/fpls.2022.1010458)
Supplement: Supplementary Table 1 — Amount of litter addition (g.100 g soil−1) in experiment 2. [file Table_1.docx]

SUPPLEMENTARY TABLE 1

Amount of litter addition (g.100 g soil^−1^) in experiment 2.

| Treatments | 10-year-old | 25-year-old | 50-year-old |
| --- | --- | --- | --- |
| Control | 0 | 0 | 0 |
| Litter addition | 0.307 | 0.549 | 1.164 |
