# Supplementary material for: Phosphorus dynamics in litter–soil systems during litter decomposition in larch plantations across the chronosequence
Source: Front Plant Sci. 2022 Oct 7;13:1010458. doi: 10.3389/fpls.2022.1010458 (PMC9585294; doi:10.3389/fpls.2022.1010458)
Supplement: Supplementary Table 2 — Importance and significance level of environmental factors in experiment 2. [file Table_2.docx]

SUPPLEMENTARY TABLE 2

Importance and significance level of environmental factors in experiment 2.

| Soil environmental factors | Explanations/% | Contribution/% | *Pseudo-F* | *P* |
| --- | --- | --- | --- | --- |
| TP | 39.6 | 65.6 | 69.6 | 0.002 |
| MBP | 15.9 | 26.4 | 37.6 | 0.002 |
| APA | 2.2 | 3.7 | 5.5 | 0.012 |
| C/N | 2.2 | 3.6 | 5.6 | 0.004 |
| TN | 0.4 | 0.6 | 0.9 | 0.374 |
| TC | < 0.1 | 0.2 | 0.2 | 0.838 |

Abbreviations: TP, total phosphorus; MBP, microbial biomass P; APA, acid phosphatase activity; TC, total carbon; TN, total nitrogen; C/N, total carbon/total nitrogen.
