# Supplementary figures and images for: Phosphorus dynamics in litter–soil systems during litter decomposition in larch plantations across the chronosequence
Source: Front Plant Sci. 2022 Oct 7;13:1010458. doi: 10.3389/fpls.2022.1010458 (PMC9585294; doi:10.3389/fpls.2022.1010458)

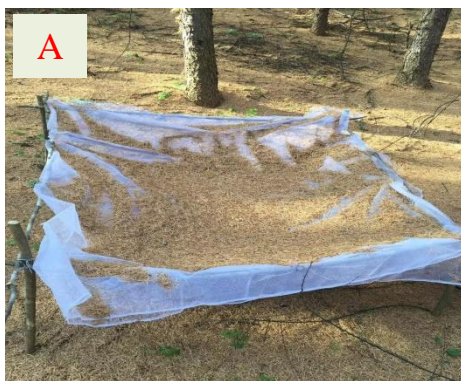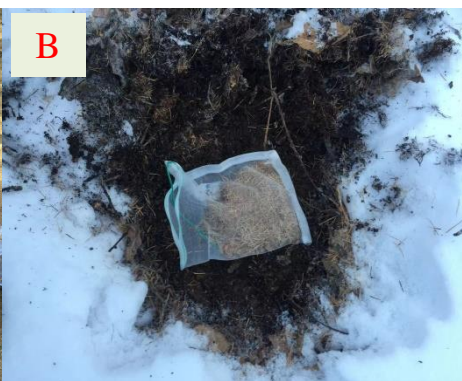

Supplement: Supplementary Figure 1 — Litter collection (A) and litterbag experiment (B). [file Image_1.pdf]
